# Consistent role of Quaternary climate change in shaping current plant functional diversity patterns across European plant orders

**DOI:** 10.1038/srep42988

**Published:** 2017-02-23

**Authors:** Alejandro Ordonez, Jens-Christian Svenning

**Affiliations:** 1 Section for Ecoinformatics and Biodiversity, Department of Bioscience, Aarhus University, Ny Munkegade 114, DK-8000 Aarhus C, Denmark

## Abstract

Current and historical environmental conditions are known to determine jointly contemporary species distributions and richness patterns. However, whether historical dynamics in species distributions and richness translate to functional diversity patterns remains, for the most part, unknown. The geographic patterns of plant functional space size (richness) and packing (dispersion) for six widely distributed orders of European angiosperms were estimated using atlas distribution data and trait information. Then the relative importance of late-Quaternary glacial-interglacial climate change and contemporary environmental factors (climate, productivity, and topography) as determinants of functional diversity of evaluated orders was assesed. Functional diversity patterns of all evaluated orders exhibited prominent glacial-interglacial climate change imprints, complementing the influence of contemporary environmental conditions. The importance of Quaternary glacial-interglacial climate change factors was comparable to that of contemporary environmental factors across evaluated orders. Therefore, high long-term paleoclimate variability has imposed consistent supplementary constraints on functional diversity of multiple plant groups, a legacy that may permeate to ecosystem functioning and resilience. These findings suggest that strong near-future anthropogenic climate change may elicit long-term functional disequilibria in plant functional diversity.

Species diversity displays distinct large-scale geographic patterns, which in turn are controlled by both historical and contemporary environmental conditions. The European flora provides one of the best-known examples of how current species distributions and richness patterns are strongly affected by not only contemporary climatic conditions^1^,^2^, but also environmental changes linked to late Quaternary glacial-interglacial climate change^3^,^4^,^5^. These historical legacies reflect both deep-time extinctions^6^,^7^, and a more recent impact of Pleistocene glaciations on European environments^8^ and biota^9^,^10^. The effects of contemporary environmental conditions on functional diversity (FD – range and variability in the trait composition of a species assemblage or region^11^) have been studied at large spatial scales^12^,^13^,^14^. However, the relative importance of historical climates in shaping current FD remains mostly unexamined, but see refs 6, 10 and 15, 16, 17 for efforts in this direction. Such lack of knowledge is astounding, given the importance of FD for ecosystem functioning^18^,^19^ and services^20^, in addition to the role of FD as a determinant of how species, communities, and ecosystems respond to changing environmental conditions^20^,^21^,^22^.

There are several ways through which FD patterns may be affected by historical climatic conditions such as the late-Quaternary glacial-interglacial climatic oscillations. First, historical climatic conditions might filter out physiologically or ecologically unsuitable species from either the local assemblage or the metacommunity species pool based on their functional attributes^10^,^23^,^24^, resulting in local functional assemblages not representing the full array of viable attributes given current environmental conditions^15^. Second, the location of glacial refugia^8^,^25^ has significant implications for recolonization dynamics in response to postglacial warming^1^,^3^,^10^,^26^, which in the case of Europe has shaped local§ species composition via colonization lags, with stronger effects at greater distances from refuge locations^1^,^5^. Third, changes in diversification rates^27^ might modify the regional assemblage of functional traits via environmentally driven selection, resulting in the emergence of new attribute states or combinations. Hence, past climatic changes such as glacial-interglacial climatic oscillations have the potential to impose lasting effects on diversity patterns. Specifically, past climatic changes have the potential to determine the size and structure of the locally realized functional trait space from the expectation under the current environmental conditions^15^.

The underlying idea of this study is that long-term climate dynamics may have affected in a similar way the functional diversity of multiple phylogenetically independent components of the European flora. The working hypotheses of this study are: (1) Geographical patterns in functional diversity will be consistent across phylogenetically independent functional groups; (2) the effects of historical climate change over the last 21,000 years as an important co-determinant of functional richness (F_Rich_ – size or range of the trait space^28^,^29^) and functional dispersion (F_Disp_ – packing of the trait space^30^) should be seen consistently across phylogenetically independent groups. The reasoning behind this hypothesis is that climatically stable and suitable conditions promote a wider coverage of the viable trait space^24^ and a more uniform functional differentiation among concurring species^24^,^31^. In contrast, areas that have experienced long-term patterns of climatic instability and were climatically unavailable during the Last-Glacial Maximum (LGM; ~21,000 years ago), would more likely experience a non-random removal of functional combinations (e.g., cold-sensitive species). Such conditions would increase the functional differences between surviving and immigrating species, resulting in smaller and scattered functional spaces.

Evidence for associations between historical environmental conditions and FD is, for the most part, indirect and restricted to a handful of studies of the European^5^,^9^,^15^ and New World flora^13^,^32^,^33^. These studies propose how historical climatic effects may act on FD via local species filtering^5^,^9^,^34^ trough climate-tolerance related traits^9^, migration lags^5^,^26^,^35^, or by inducing the functional differentiation of regional species pools^31^. This study aims to assess the influence of historical climatic conditions by answering two previously unexamined questions: (1) *Do plant FD geographic patterns of multiple evolutionary independent European plant groups (orders) exhibit prominent late-Quaternary glacial-interglacial climate change imprints?* (2) *How does the importance of historical environmental conditions compare to that of contemporary factors across multiple evolutionary independent European plant groups (orders)?* To answer these questions, the geographic patterns in F_Rich_ and F_Disp_ for six widely distributed and species rich orders of European angiosperms were estimated, by combining data on species distributions from the Atlas Florae Europaeae^36^ with trait information. Then, using spatial autoregressive models (SAR) and information theory approaches (see *Methods*), we evaluated the link between contemporary FD patterns and both late-Quaternary glacial-interglacial climate change (here measured via climate velocity; see *Methods*) and proximity to glacial refugia (here measured as accessibility; see *Methods*), while accounting for contemporary environmental effects.

The results presented here show that macro-scale, functional diversity geographic patterns for multiple European plant groups were indeed associated with late-Quaternary glacial-interglacial climate change even though the end of the last glaciation occurred ~11,500 years ago. These results indicate that future climate change trends may elicit not only short-term shifts in ecosystem functioning, and potentially long-term functional disequilibria. Consequently, understanding the impact and generality of these historically determined patterns acting in conjunction with contemporary environmental conditions is paramount to developing, particularly for the development of accurate predictive methods for ecosystem function and service responses to future climatic changes.

## Results

**Figure 1:**
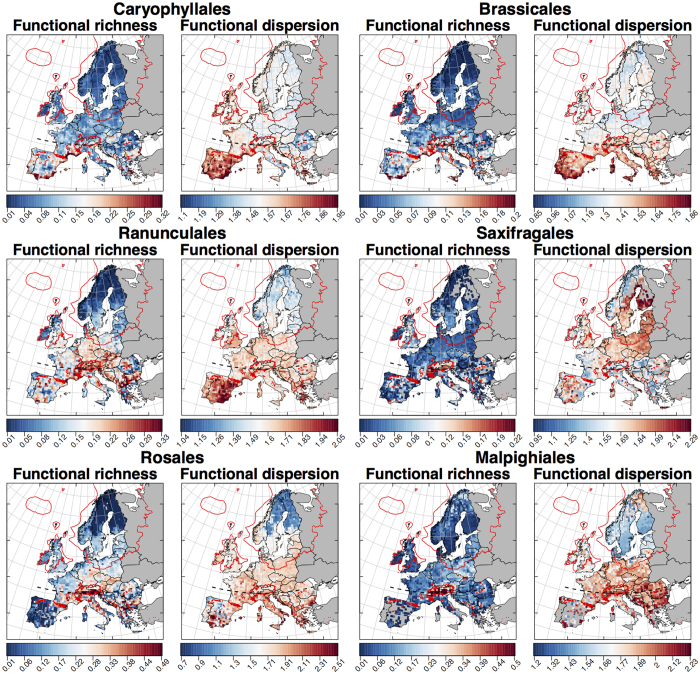
The functional diversity of six European plants orders for each of the Atlas Flora Europaeae grid cells (~50 km × 50 km). Values show the functional richness and dispersion of the trait space described by all species within an order occurring at a site. Represented values are the per-grid means of functional diversity estimates coming from ten different imputed datasets (*see Methods*). The dashed black line represents the 46°N latitude, which marks an estimate of the maximum northern limit of temperate tree full-glacial refugia^1^,^25^. Red delimited areas show the maximum extent of the ice sheet 21,000 years ago based on ref. 59. Groups ordered from top-to-bottom and left-to-right according to decreasing species richness. Figures generated using R^57^ version 3.0.2 https://www.r-project.org based on mean functional diversity estimates across imputed datasets for each Atlas Flora Europaeae grid cells.

**Figure 2:**
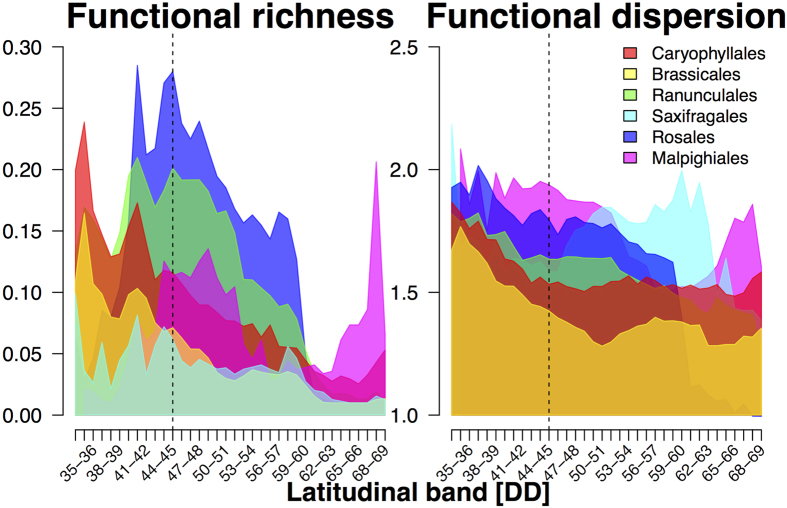
Latitudinal gradients in functional diversity for six orders of European angiosperms. Distributions show the functional richness (left) and dispersion (right) of the trait space described by all species within each of the six evaluated orders for 1-degree latitudinal bands. Represented values are the per-grid mean from ten functional diversity estimates (*see Methods*). Assessed orders are illustrated with different colours. Dashed black line represents the 46°N latitude, which marks an estimate of the maximum northern limit of temperate tree full-glacial refugia^1^,^25^.

Using multi-trait metrics, we estimated the geographic patterns in F_Rich_ and F_Disp_ for species assemblages from six orders of European angiosperms (Fig. 1). As expected, F_Rich_ and F_Disp_ per-grid values differed among orders, a pattern confirmed by the significant better fit of a random intercept model when compared to a model without a random effect (p < 0.001 for both F_Rich_ and F_Disp_ Log-likelihood ratio tests). Regardless of the differences in F_Rich_ and F_Disp_ per-grid values across the evaluated orders, and in agreement with our hypotheses, spatial patterns, and latitudinal gradients were consistent across orders (Figs 1 and 2). F_Rich_ increased from Southern to Central Europe and decreased from Central to Northern Europe (Figs 1 and 2a). F_Rich_ maximum values were recorded across Central Europe, notably coinciding with the southern band of mountainous areas (Fig. 1). F_Disp_ decreased in a south-to-north direction (Figs 1 and 2b), but with high-values in both the Italian and Balkan peninsulas, and northern Sweden and Finland.

Dutilleul’s corrected correlation tests indicate a positive association between F_Disp_ and F_Rich_ (*ρ* ranging between 0.46 and 0.64 across orders, and p < 0.05 in al cases). Association with species richness was significant for F_Rich_ (*ρ* ranging between 0.67 and 0.9 across orders, and p < 0.05 in al cases), and non-significant for F_Disp_ (*ρ* ranging between 0.1 and 0.34 across orders, and p ≥ 0.05 in al cases). Although there is a strong relationship between F_Rich_ and local species richness, observed values consistently differ from those expected solely on species richness, and these relationships quickly saturate as the number of species increases (as described in the Supplementary material). These results demonstrate that local F_Rich_ consistently deviates from expectations based on taxon richness alone, particularly in areas with high species richness.

No single trait was the sole determinant of the observed FD patterns, as indicated by spatial models evaluating the association between range and variability of evaluated traits with F_Rich_ and F_Disp_ respectively (as described in the Supplementary information). Nonetheless, single and multiple regressions between FD and trait richness and dispersion show that canopy height and seed weight, which are both related to dispersal capacity, exhibit the strongest associations with F_Rich_ and F_Disp_.

**Figure 3:**
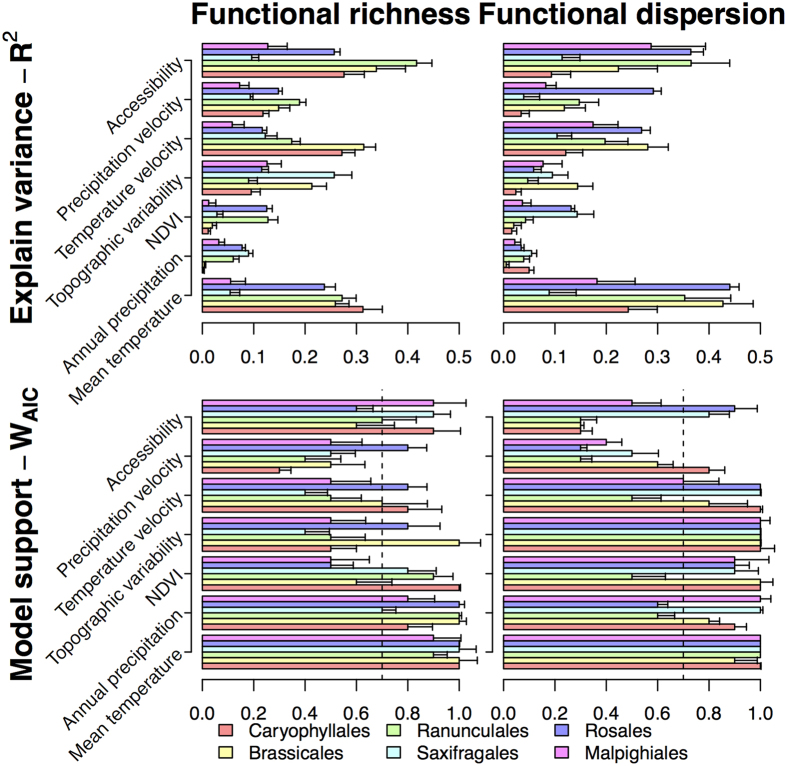
Explained variance (top row) and relative model support (bottom row) of historical and contemporary environmental factors. The bar height shows the mean, and whiskers the variability, in explained variances of single-predictor models (Pseudo-R^2^) or mean relative model support (*W*_*AIC*_) for the evaluated predictors for each of the six orders included in this study across all ten imputed datasets (*see Methods*). Dashed vertical lines in the bottom panels indicate the 70% *W*_*AIC*_ cut-off point.

**Figure 4:**
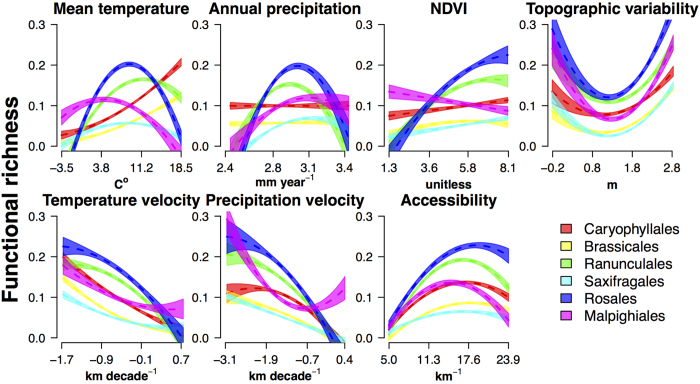
Changes in functional richness of six European plant orders as a function of contemporary and historical environmental conditions. Plots show the effects of mean annual temperature, total annual precipitation NDVI, topographic heterogeneity, temperature velocity, precipitation velocity and accessibility on functional richness. Assessed orders are illustrated with different colours. Shaded areas over the dashed regression lines represent the 95% confidence interval of predicted values for each evaluated order.

**Figure 5:**
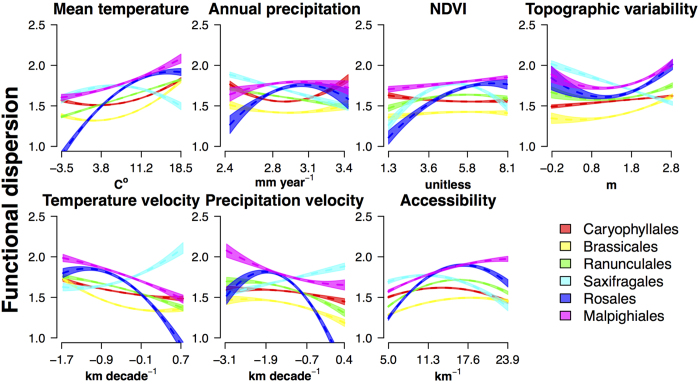
Changes in functional dispersion of six European plant orders as a function of contemporary and historical environmental conditions. Plots show the effects of mean annual temperature, total annual precipitation NDVI, topographic heterogeneity, temperature velocity, precipitation velocity and accessibility on functional dispersion. Assessed orders are illustrated with different colours. Shaded areas over the dashed regression lines represent the 95% confidence interval of predicted values for each evaluated order.

The explained variance (Fig. 3 top) and shape of the relation of FD with evaluated environmental predictors (Figs 4 and 5) showed a wide variability across evaluated orders. Nonetheless, some generalities emerge regarding the main drivers of F_Rich_ and F_Disp_ change in the region. Overall, mean annual temperature and topographic variability were the predictors with the highest explained variance for both F_Rich_ and F_Disp_. Furthermore, F_Rich_ (Fig. 4) and F_Disp_ (Fig. 5) increased with mean annual temperature, annual precipitation, and productivity. However, the explained variance of single-predictor models (Fig. 3 top) shows that for almost all the evaluated orders, variability in F_Rich_ and F_Disp_ was also strongly related to at least one of two historical variables: late-Quaternary glacial-interglacial temperature velocity and accessibility to glacial refugia. The observed directionality of associations with historical climate aligned with our expectations of the effects of historical variables on functional diversity (Figs 4 and 5). Specifically, F_Rich_ and F_Disp_ decreased as temperature velocities increase (high instability), and increased with proximity to refugia (greater accessibility to post-glacial recolonization).

For a more comprehensive assessment of the relative importance of each of the evaluated predictors across evaluated orders, Akaike weights (W_AIC_) were used to determine which predictors had the highest support as explanatory factors of F_Rich_ and F_Disp_. For this purpose, starting with a pool of 576 possible SAR models for each one of the evaluated orders, the W_AIC_ values for all the models including the predictor of interest were added (see *Methods* for details). The evaluated models described all non-interacting linear and unimodal (linear + quadratic responses) combinations of historical and contemporary predictors. Across evaluated orders, contemporary predictors had higher relative importance (highest W_AIC_) when compared to historical predictors (Fig. 3 bottom). The contemporary predictors showing the highest relative importance scores for F_Rich_ included mean annual temperature and topographic heterogeneity, closely followed by annual precipitation and NDVI (Fig. 3). In the F_Disp_ case, mean annual temperature and annual precipitation were the variables with W_AIC_ values consistently above 70% (Fig. 3).

The two historical predictors that consistently showed importance scores comparable to those of contemporary predictors were temperature velocity and postglacial accessibility (Fig. 3 bottom). Across all the evaluated orders, at least one of these two historical predictors showed W_AIC_ scores above 70% for either F_Rich_ or F_Disp_. As indicated by the W_AIC_ scores, the importance of precipitation velocity is relatively lower, as it ranged across evaluated orders between 1% and 30% for F_Rich_, and between 33% and 76% for F_Disp_ (Fig. 3).

Model average regression coefficients for the evaluated orders’ SAR regressions (Tables 1 and 2) provide further support for the general importance of historical climate, either in the form of temperature stability or accessibility, as a key determinant of the FD. Magnitudes of averaged standardized coefficient show significant effects on FD of both historical and contemporary factors in all the evaluated orders (Figs 4 and 5; Tables 1 and 2). Specifically, F_Rich_ and F_Disp_ increased with energy availability, topographic heterogeneity, accessibility to glacial refugia, and late-Quaternary temperature stability, (Figs 4 and 5; Tables 1 and 2). Overall, postglacial accessibility and late-Quaternary temperature velocity act in conjunction with mean annual temperature and topographic heterogeneity as the main determinants of F_Rich_ for most orders (Table 1). Similarly, accessibility, mean annual temperature, and annual precipitation were particularly influential in shaping observed F_Disp_ patterns for most orders (Table 2).

## Discussion

This study determined the FD geographic patterns of multiple phylogenetically independent groups of European plants by combining species distribution data and trait information. The relations of these geographic patterns with multiple environmental predictors show that historical climates and contemporary environments shape the FD patterns of European plants. The strong association of contemporary F_Rich_ and F_Disp_ with Quaternary glacial-interglacial climate change is surprising, given that current abiotic environmental conditions^18^ in conjunction with trait-based assembly rules^37^ are often considered to be the main drivers of continental FD patterns.

Both F_Rich_ and F_Disp_ exhibited geographic patterns wherein maximum values were in the main known glacial refugia for non-boreal temperate species in Southern Europe (notably, Italian and Balkan peninsulas^38^). Large F_Rich_ and F_Disp_ values were also in areas of high topographic variability (Pyrenees and Alps) that provide a buffer against climatic variations, and in regions that are highly accessible to postglacial colonization^2^,^5^. The tendency towards high F_Rich_ and F_Disp_ in areas know to be glacial refugia and have a wide elevation heterogeneity was generalized across evaluated orders.

The continental-level assessment of historical climatic effects on European F_Rich_ and F_Disp_ presented here showed the generality of the strong complementary effects of historical and contemporary drivers. The fact that such effects were consistent across all six evaluated orders highlights their joint importance in determining regional F_Rich_ and F_Disp_ patterns, even across evolutionally independent lineages. Support for the importance of historical factors as determinants of contemporary FD comes from the fit of regression models (pseudo–R^2^), the comparable support of contemporary and historical predictors (W_AIC_), and the size of SAR model averaged regression coefficients across evaluated orders.

The reported relationships between geographic patterns of European plant F_Rich_ and F_Disp_ and historical climatic conditions show that the constraints imposed by late-Quaternary glacial-interglacial climate change on species current ranges also influence the FD geographic patterns observable today. Moreover, these historical effects constitute a general trend across multiple evolutionary independent groups of European plants. Nonetheless, the subtle differences in the geographic patterns observed across the evaluated orders may be attributable to the spatial variability in historical species sorting by glacial-interglacial climate changes. Other possible factors driving the observed differences between groups are differences in dispersal capacity across groups of organisms^4^,^5^,^26^,^32^,^35^, and the differences in tolerance to historical changes in precipitation and temperature^32^ of evaluated orders.

The directions of the association between F_Rich_ and F_Disp_ and historical conditions were consistent with the predictions of a higher F_Rich_ and a lower F_Disp_ in areas with stable climatic conditions and proximity to glacial refugia. The consistency in the reported associations and the current knowledge of the vegetation history of this region provide some indications of how the observed influence of historical factors might be the result of multiple interacting mechanisms. Such mechanisms include dispersal-limited recolonization dynamics in climatically suitable areas^3^,^4^,^5^, the historical filtering of physiologically or ecologically unsuitable functional characteristics from the regional metacommunity pool^23^,^24^, the geographic location of glacial refugia^8^,^25^, and non-random community assembly mechanisms (e.g., species-level priority effects^39^).

The historical effects on F_Rich_ are consistent with the tendency for past climatic conditions to prune trait spaces via mechanisms altering the number and types of organisms within a location (such as non-random extinctions or re-colonization^9^ and dispersal lags^5^). Similarly, F_Disp_ is mainly positively associated with postglacial accessibility, which again may reflect how both the non-random pruning of the functional space based on attributes related to dispersal ability, the existence of multiple viable trait combinations under mesic conditions, and a homogenization of the spacing among surviving or re-immigrated species from refugia areas. As a result, current FD values of evaluated orders can be considered to be in disequilibrium with contemporary climate; a result aligned with the reported disequilibrium in other diversity components in Europe^2^,^5^ and North America^32^,^40^.

The influence of the contemporary environment on FD illustrates the known effect of recent environmental conditions as key determinants of species distribution and richness patterns among plants in Europe^1^,^3^ and other regions^13^,^37^,^41^,^42^. As expected, contemporary mild climates, high levels of productivity, and large topographic heterogeneity exhibited positive effects on F_Rich_ and F_Disp_. However, the strength of these effects varied between evaluated orders and evaluated aspects of the functional space. The relation between F_Rich_ and both mean annual temperature and topographic heterogeneity shows how contemporary environmental conditions can determine the possible functional space size that an assembly can occupy^29^. Similarly, the relationship of F_Disp_ to mean annual temperature and annual precipitation are consistent with the tendency of areas with suitable and productive climates to show evenly packed functional spaces. Such conditions reduce the strength of environmental filtering^31^, allowing both functionally distinct species with similar habitat requirements and functionally similar species with different habitat requirements to coexist within a region, e.g., via small-scale habitat partitioning.

The results presented here provide four strong arguments supporting the relevance of historical climatic legacies as determinants of FD patterns across multiple evolutionary independent groups of European angiosperms: (1) The inability of both multiple and individual trait models solely based on contemporary climates to account for most of the variation in F_Rich_ and F_Disp_, (2) the significant association between historical predictors with F_Rich_ and F_Disp_, (3) the comparable importance of contemporary and historical predictors, and (4) the generality of these patterns across multiple evolutionarily independent groups.

In summary, this study shows that historical climate stability and distance to refuge locations influence the geographic distribution of FD in multiple evolutionary independent European plant groups. The results thereby also indicate that local F_Rich_ and F_Disp_ levels are not necessarily optimized to the contemporary environment, but may instead be in disequilibrium due to the occurrence of non-random extinctions^9^, migration lags^5^, and possibly also species-level priority effects^39^. The findings imply that local and regional F_Rich_ and F_Disp_ losses due to future climate change trends or other anthropogenic pressures may have long recovery times. Moreover, the results of this study suggest that future climate change will not only elicit short-term shifts in ecosystem functioning but likely will also induce long-lasting ecosystem level effects, strengthened by long-term functional disequilibria. The underlying mechanisms behind such historical effects need to be evaluated in more detail, notably to facilitate incorporating them into predictive models of future dynamics of vegetation and related ecosystem functioning to enhance the accuracy of climate-change impact and dynamics estimations.

## Methods

Plant species distribution data for this study comes from the Atlas Florae Europaeae (AFE), which maps the distribution of European flora on an equal-area mapping unit of ~50 × 50 km (AFE cells^36^) based on the Universal Transverse Mercator (UTM) projection and Military Grid Reference System (MGRS). Observations for the former Soviet Union were not included in the analyses due to uncertainties in the records for this area. Selected AFE grid cells were the geographic units for all the computations and analyses done in this study.

Analyses in this study focus on the distributions of six of the most species-rich, widely distributed, and functionally distinct Angiosperm orders contained in the AFE: Caryophyllales (1384spp), Brassicales (973spp), Ranunculales (654spp), Saxifragales (352spp), Rosales (334spp) and Malpighiales (73spp). The use of orders as independent units of analysis provides the benefit of replicability among groups with an independent evolutionary history, which would translate to their trait values and composition. As a result, any similarity in the observed geographic trends and the effects of contemporary and historical environmental conditions on the regional functional diversity (FD) patterns can be attributed to the similarity in the mechanisms generating the observed pattern across multiple groups, and not to the taxonomic composition of the evaluated species pool. The regional coverage and species richness for each order are presented as Supplementary information.

Five ecomorphological traits were used to describe the functional space defined by coexisting species of the same order. Used traits are specific leaf area (SLA, cm^2^*g^−1^), maximum stem height (H_max_, m), seed mass (SWT, mg), stem specific density (WD, kg*m^−3^), and growth form (aquatic, herb, graminoid, shrub, tree, or vine). These traits were selected due to their usefulness for distinguishing functional strategies among concurring plants^43^,^44^,^45^, the known link between these traits and abiotic environments^44^, and the common use of these traits for predicting the geographic distribution of vegetation types, ecosystem functions, and regional mean plant physiological response assessment.

Mean trait values were initially determined based on multiple trait databases, described in the Supplementary information. Gaps in the database were filled using an evolutionary (based on genus), and morphological (based on growth forms) restricted Multivariate Imputation Chained Equations procedure (MICE; ref. 46), using a dataset that covers all the species with known traits in the AFE. The MICE approach has been shown to provide accurate trait estimates and conserves allometric relations when up to 60% of data is missing^47^. On average, between 40 and 50% of the evaluated species required the imputation of a trait. The imputation procedure starts with an observed, incomplete data set for which a multivariate distribution for the attribute of interest is defined based on the available information, and then used to drawing an imputation of the missing values using Markov Chain Monte Carlo (MCMC) techniques. Multiple imputed values are generated for each empty case (ten in total), by randomly selecting values for the missing observation from a simulated trait space. For more details on the methodology see refs 15, 46 and 48. Since this approach produced ten different trait estimates for each gap in the database, the estimation of functional diversity and regressions analyses used each of the imputed replicates independently to account for the uncertainty in the trait imputation procedure. Mean trait values per AFE grid for all four continuous traits are presented as Supplementary information.

For each order and imputed dataset, two complementary FD metrics were estimated: functional richness (F_Rich_) and functional dispersion (F_Disp_). The selected metrics describe two aspects of a species assemblage multivariate trait composition. F_Rich_ measures the size of the functional space circumscribed by the species in an assemblage based on the volume of a multivariate convex hull^29^. F_Disp_ measures the packing of the functional space described by the species in an assemblage based on the distance to the mean trait composition of the community^30^. The FD metrics were evaluated using continuous traits (SLA, H_max_, SWT, and WD). Before the analyses, traits were log_10_ transformed as they were log-normally distributed and standardized (mean = 0 and SD = 1) to ensure that all traits contribute equally and that the units used to measure traits have no influence on the FD estimation^28^.

A total of seven predictors were selected to summarize historical and contemporary environmental conditions. The variables chosen as predictors summarize alternative ecological mechanisms that are postulated as factors determining diversity patterns at continental and global scales (see refs 5, 10 and 49, 50, 51). Spatially corrected pairwise correlations between predictors ranged from −0.25 to 0.25; this indicates that there are no clear patterns in the between predictors correlations. For a graphical representation of the evaluated and the correlation among predictors see the Supplementary information.

Used variables were divided into historical and contemporary predictors. Historical predictors include climatic stability^10^ and accessibility to suitable environments^5^,^10^ at the end of the Last Glacial Maximum (LGM; ~21,000 yrs ago). We focus on environmental changes since the LGM climate changes during this period are relatively well known. Also, by focusing on this period, we can focus on evaluating the effects of colonization dynamics and local extirpations without having to consider regional speciation and extinction events. Historical climatic stability was measured as the annual temperature and precipitation velocities (measured in kilometers × decade^−1^) from the LGM. As in ref. 52, climate velocities were calculated as the ratio between temporal anomalies (that is the absolute difference between the LGM and current temperature and precipitation) and the spatial gradients (variability in temperature and precipitation during the LGM on a 3 × 3 grid cell neighbourhood). We used climate velocity as a measure of stability as it captures both the changes in climatic conditions over time (temporal anomaly) and the buffering effect of the surrounding area (spatial heterogeneity). Following the approach described in ref. 2, accessibility to postglacial re-colonization from ice-age refugia (measured in kilometers^−1^) was estimated as the sum of inverse distances between an AFE grid cell and regions considered to be suitable for cool-temperate trees during the LGM according to ref. 25 specifications. Historical climate conditions were determined based on three different climatic models (CCSM, MIROC, and MPI) for the last glacial maximum from the Paleoclimate Modelling Intercomparison Project Phase II at a resolution of 5 × 5 arc-minutes, and summarized as the mean within each AFE cell. We focused on both climatic stability and accessibility to refugia as these provide an estimate of the effects of the magnitude of change driving local extirpations and colonization distance for suitable areas at the end of the LGM.

Contemporary predictors describe water–energy dynamics^49^,^50^ and the importance of spatial heterogeneity^51^ as determinants of diversity patterns at continental scales. Climatic predictors were obtained from refs 53 and 54 and included the mean annual temperature, total annual precipitation (mm × year^−1^). Normalized Difference Vegetation Index (NDVI, unitless) was obtained from ref. 54. Habitat heterogeneity (variability in elevation measured in m, estimated using ref. 53 elevation model). Annual precipitation, habitat heterogeneity and glacial temperature and precipitation velocities show a lognormal distribution, so they were log_10_-transformed before the statistical analyses. Like historical variables, each of the contemporary factors was summarized as the mean within each AFE cell.

A spatial simultaneous autoregressive error modelling approach (SAR) was used to account for spatial autocorrelation in the data. In all cases, FD was the response variable, and environmental predictors (linear or unimodal responses) were used as explanatory variables. The spatial weights matrices in the SAR models were determined as the first neighbour of each site.

For each order, the relative support of each predictor in isolation was determined based on model fits of single-predictor models, and information theoretical approaches based on multiple- predictor models. The variance explained by each historical and contemporary predictor in isolation was assessed based on the model fit of single-predictor SAR models, determined using Nagelkerke pseudo-R^2^ values^55^. Additionally, the relative importance of each historical and contemporary predictor was established by summing the Akaike weights (*W*_*AIC*_; calculated following ref. 56) for all of the models in which the predictor of interest was included. W_AIC_ were determined based on all 576 possible SAR models based on the combination of historical and contemporary predictors either as linear or unimodal responses. Last, the relative effect of historical and contemporary predictors was determined using the model averaged regression coefficients^56^. For this, the standardized regression coefficients were averaged across all of the 576 evaluated models evaluated for an order-imputation combination, weighting each value by the *W*_*AIC*_ for the model that contained it. All statistical analyses were conducted using R^57^ version 3.0.2, and spatial analyses were conducted using the R library spdep^58^ version 0.5–68.

## Additional Information

**How to cite this article:** Ordonez, A. and Svenning, J.-C. Consistent role of Quaternary climate change in shaping current plant functional diversity patterns across European plant orders. *Sci. Rep.* **7**, 42988; doi: 10.1038/srep42988 (2017).

**Publisher's note:** Springer Nature remains neutral with regard to jurisdictional claims in published maps and institutional affiliations.

## Supplementary Material

Supplementary Text

## Figures and Tables

**Table 1:** Contemporary and historical model-averaged regression coefficients for models explaining functional richness for six orders of European angiosperms.

Functional richness							
		Caryophyllales (1384)	Brassicales (973)	Ranunculales (654)	Saxifragales (352)	Rosales (334)	Malpighiales (73)
Intercept	*a*	0.136	0.024	0.147	−0.024	−0.03	−0.129
Annual Temperature	*b*	0.271	0.047	0.29	−0.395	0.448	−0.77
	*b*^*2*^	0.001	−0.087	−0.572	−0.611	−0.784	
Annual Precipitation	*b*	0.001	0.023	−0.02	0.135	−0.011	0.128
	*b*^*2*^	0.006					0.002
NDVI	*b*	−0.15	0.217	0.017	0.189	0.076	0.538
	*b*^*2*^	−0.001	−0.29	−0.035	−0.096		−0.559
Topographic heterogeneity	*b*	−0.106	0.029	0.112	0.006	0.194	−0.128
	*b*^*2*^	0.216	0.113		0.126		0.228
Annual temperature Velocity	*b*	−0.103	−0.057	−0.02	−0.131	−0.131	−0.043
	*b*^*2*^	0.019	0.012				
Annual Precipitation Velocity	*b*	0.002	0.03	0.007	0.021	−0.003	−0.014
	*b*^*2*^	−0.043	−0.004				
Accessibility	*b*	−0.024	0.003	0.029	−0.344	0.304	−0.117
	*b*^*2*^	−0.002	−0.001	−0.001			−0.054

Average regression coefficients estimated based on the regression coefficients from 576 spatial autoregressive models (SAR) using ref. 56 model averaging approach. Estimates show the mean functional diversity (*a*), and both the lineal (*b*) and quadratic (*b*^*2*^) modelled averaged standardized coefficients for each evaluated predictor. Empty cell are model modelled averaged standardized coefficients lower than 0.001. Values in parenthesis at the top of every column show the number of evaluated species per order.

**Table 2:** Contemporary and historical model-averaged regression coefficients for models explaining functional dispersion for six orders of European angiosperms.

Functional dispersion							
		Caryophyllales (1384)	Brassicales (973)	Ranunculales (654)	Saxifragales (352)	Rosales (334)	Malpighiales (73)
Intercept	*a*	1.733	1.147	2.456	1.888	0.569	2.012
Annual Temperature	*b*	0.314	0.087	0.125	0.731	0.31	−0.16
	*b*^*2*^	0.09	0.13	−0.133	−0.668	−0.029	0.003
Annual Precipitation	*b*	−0.107	0.082	−0.349	−0.011	0.119	−0.223
	*b*^*2*^	0.156		0.194	0.001	−0.059	0.277
NDVI	*b*	−0.176	0.022	−0.068	0.131	0.061	0.102
	*b*^*2*^	0.001	−0.035	−0.006	−0.052	−0.066	−0.086
Topographic heterogeneity	*b*	0.006	−0.056	0.034	−0.016	0.049	−0.095
	*b*^*2*^	0.019	0.158	−0.008			0.11
Annual temperature Velocity	*b*	−0.062	−0.04	−0.019	0.011	−0.049	−0.023
	*b*^*2*^	0.002	0.009				0.004
Annual Precipitation Velocity	*b*	0.002	−0.009	−0.016	0.026	−0.046	0.016
	*b*^*2*^	−0.001	−0.024			−0.004	−0.007
Accessibility	*b*	−0.067	−0.092	0.501	−0.445	0.183	0.516
	*b*^*2*^	−0.211	−0.017	−0.253		−0.049	−0.142

Average regression coefficients estimated as in Table 1. Empty cell are model modelled averaged standardized coefficients lower than 0.001. As in Table 1, values in parenthesis at the top of every column show the number of evaluated species per order.
