# Supplementary material for: Consistent role of Quaternary climate change in shaping current plant functional diversity patterns across European plant orders
Source: Sci Rep. 2017 Feb 23;7:42988. doi: 10.1038/srep42988 (PMC5322331; doi:10.1038/srep42988)
Supplement: Supplementary Text [file srep42988-s1.pdf]

Supporting Information

Consistent role of Quaternary climate change in shaping current plant functional diversity patterns across European plant orders

Alejandro Ordonez[1] And Jens-Christian Svenning[1]

[1]Section for Ecoinformatics and Biodiversity, Department of Bioscience, Aarhus University, Ny Munkegade 114, DK-8000 Aarhus C, Denmark

---

Supporting Information Overview

This document provides supplementary information not contained in the main text of the article "*Consistent role of Quaternary climate change in shaping current plant functional diversity patterns across European plant orders.*" ***SI Text-1*** provides an extended description the used data (distribution and traits), the trait imputation procedure, functional diversity estimates, and the predictors used in the statistical analyses presented in the main text. ***SI Text-2*** describes the association between multivariate metrics of functional diversity with both species richness and univariate measurements of trait richness and dispersion. It also discusses the difference between observed and expected values of functional diversity based on the local species richness.

***SI Text-1*. Extended methods**–Description of the used traits, the trait imputation procedure, functional diversity estimates, and statistical analyses

Distribution data.

This work uses plant species distribution data from Atlas Florae Europaeae (AFE; ref.[1]), which maps the distribution of European flora on an equal-area mapping unit of ~50×50km (AFE cells) based on the Universal Transverse Mercator (UTM) projection and a Military Grid Reference System (MGRS). We excluded observations in the former Soviet Union due to uncertainties in these records. This study focuses on angiosperms species, contained in six orders: Caryophyllales – 1384spp, Brassicales – 973spp, Ranunculales – 654spp, Saxifragales – 352spp, Rosales – 334spp, and Malpighiales – 73spp. Plant orders included in this study compose the majority of the AFE and represent a pool of independent evolutionary lineages. For consistency with trait data, occurrences of taxonomic units below the species level (e.g., forms, varieties, and subspecies) were collapsed to those of the corresponding species. The distribution and local species richness of each of the evaluated orders in presented as **Fig. S1**.

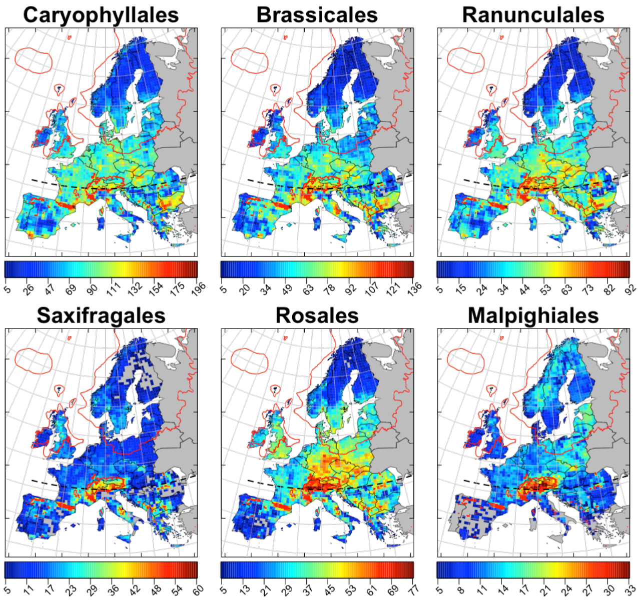

Figure S1. Species richness of the six evaluated European Angiosperm orders. Values represent the number of individual species per Atlas Florae Europaeae grid-cells (~50×50 km). Dashed line indicated the northern limit of post-glacial refugia for cold tolerance trees as summarized in ref.[2]. Figure generated using R[3] version 3.0.2. <https://www.r-project.org> based on species counts for each AFE-grid cells.

Used traits.

Five ecomorphological traits were used in the analyses presented in this study: specific leaf area (SLA, cm^2*g^{-1}), seed mass (SWT; mg), maximum stem height (Hmax; m), stem/wood density (WD; $kg*m^{-3}$) and growth form (tree, shrub, herbs/forbs, vines/lianas). These represent some of the best traits for predicting the geographic distribution of vegetation types and assessing the mean plant physiological response in a region. Used traits were also selected due to their use to discriminate between distinctive functional strategies among concurring plants[4-6] and the link between these traits and the abiotic environment[7-11]).

The SLA of a plant indicates how much light-capturing area it produces for each gram of leaf tissue. SLA is negatively correlated with leaf thickness, lifespan, toughness and sensitivity to herbivores and positively associated with mass-based measurements of leaf nitrogen content, photosynthetic capacity, plant relative growth rate, all of which are relevant to plant performance[7]. Therefore, SLA reflects a species position along the leaf economics spectrum[5,7,12]. H_{max} represents the balance between the gains from access to light, the cost of structural support (given the disturbance regime) and water transport, and the sensitivity to biomass loss from mechanical disturbances[4,5,13]. H_{max} is also a proxy for other key traits indicating plant growth[13]. SWT represents the balance between offspring number (seed production) and size (seed size), a trade-off often related to age-dependent survival probability[4,14]. SWT is also negatively correlated with dispersal distance (in the case of wind-dispersed species) and positively associated with seedling survival probability under light or water limited conditions[14,15]. Lastly, WD represents the trade-offs between growth rates, construction costs, and mortality rates[16]. Within this “wood economics spectrum”, a species falls along a continuum going from high volumetric growth rates, low construction costs, and high mortality rates, towards low volumetric growth rates, high construction costs, and low mortality rates[9,16].

Trait imputation

Mean trait values for each continuous trait were initially determined using the LEDA trait database[17] for as many species as possible. Missing trait information was obtained from multiple data sources: SLA[7,18]; maximum canopy height[10,17-19], seed mass[14,20]; woody density[16]; and growth form[21].

To "fill-in" missing values, we used a multiple-imputation approach (MICE; refs.[22,23]). It is a Markov Chain Monte Carlo (MCMC) in which the missing values are replaced by a small set of modelled alternatives (usually <10). The MICE approach is valid in those situations when a multivariate distribution is a reasonable description of the data, as in the case of trait values. MICE is often preferred over other imputation techniques as it is readily available, provides an approximate solution with good properties, and is not problem-specific. The R package mice[24] was used to generate imputed values.

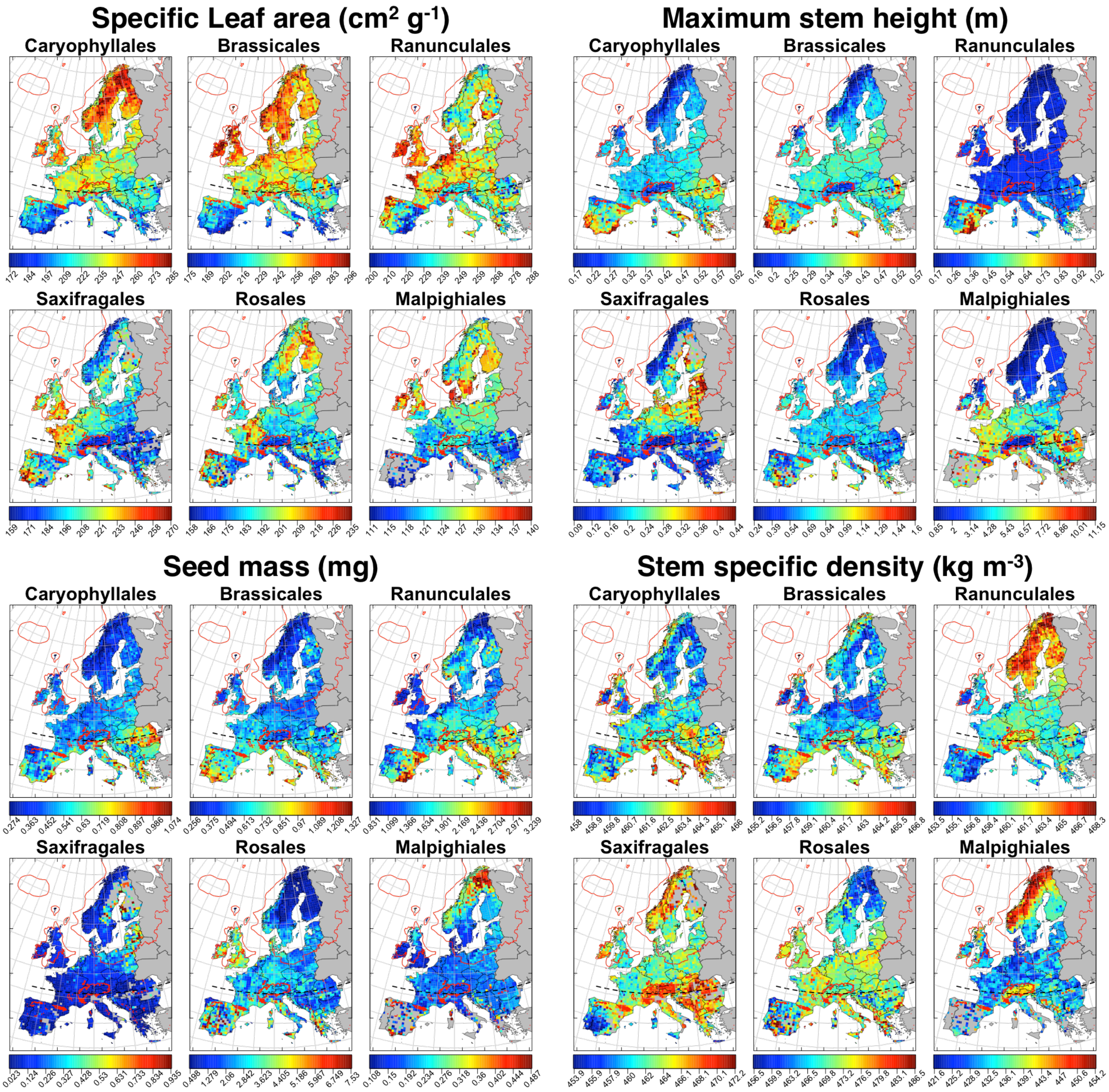

Figure S2. Mean values of four ecomorphological traits for six orders or European angiosperms. Trait values summarized as the geometric mean value of all the species co-occurring in each of the Atlas Flora Europaeae grid cells (~50×50 km). Red polygon shows the maximum extent of the ice sheet 21,000 years BP. Dashed line indicated the northern limit of post-glacial refugia for cold tolerance trees as summarized in ref.[2]. Figure generated using R[3] version 3.0.2. <https://www.r-project.org> based on estimates of mean trait-values for each AFE-grid cell.

A predictive mean matching (*pmm*) approach was used to impute missing values. The *pmm* is a semi-parametric imputation approach and the default method for continuous variables in MICE. The *pmm* approach is similar to a regression method, except that the model does not generate the imputed values, but rather serves to construct a metric for matching cases with missing data with similar cases with observed data[25,26]. Imputed values are determined by randomly sampling the observed values based on their match to the results of a simulated regression model. In other words, observations whose regression-predicted values are closest to the regression-predicted value for the missing value are used as imputed values. In our case, the implemented regression model is based on the available trait information, and both evolutionary relatedness (genus) and morphological attributes (growth form). The predictive mean matching method ensures that imputed values are plausible and might be more appropriate than a regression method if the normality assumption is violated[27]. Mean trait values for the four evaluated traits are presented in **Fig. S2**.

Functional diversity estimation

In this study, the functional composition of a site was described using two complementary functional diversity metrics: functional richness[28] and functional dispersion[29]. Range and variance from each of the evaluated traits were computed for comparisons purposes (see ***SI Text-2***). Functional richness (F_{Rich}) measures the range of the trait spectrum of a species assemblage. This measurement is based on the multivariate trait space filled by an assemblage as measured by the size of a multivariate convex hull[28]. Functional dispersion (F_{Disp}) measures the functional packing of the species assemblage. This measurement is based on the distance of individual species to the assemblage mean trait composition[29]. Functional diversity metrics were evaluated using all continuous traits. Before the analyses, all continuous traits were $\log_{10}$ transformed as they are log-normally distributed. Transformed were also standardized (mean=0 and SD=1) to ensure that all traits contribute equally to the functional diversity estimation and that the units used to measure traits have no influence in the functional diversity estimation[30].

Environmental information.

A total of seven predictors were selected to summarize historical and contemporary environmental conditions in Europe (**Table S1** and **Fig. S3**). Used predictors describe the ecological mechanisms most often consider as the determinants of diversity patterns at continental and global scales (see refs.[31-35]).

Historical predictors (**Table S1**) include climatic stability[34] and accessibility to suitable environments[34,35] at the end of the Last Glacial Maximum (LGM; ~21,000 years ago). Historical climatic stability was measured as the annual temperature and precipitation velocities (measured in kilometers×decade^{-1}) from the LGM. Velocities were calculated following ref.[36] as the ratio between temporal anomalies (that is the absolute difference between the LGM and current temperature and precipitation) and the spatial gradients (variability in temperature and precipitation during the LGM on a 3×3 cell neighbourhood). Following the approach in ref.[37], accessibility to postglacial re-colonization from ice-age refugia (measured in kilometers^{-1}) was estimated as the sum of inverse distances between an AFE grid cell and regions considered to be suitable for cool-temperate trees during the LGM. Suitability of cool-temperate trees 21,000 years BP was defined, following ref.[38], as areas where GDD ≥800°C, mean temperature of the coldest month ≥−15°C, and summer precipitation ≥50mm. Historical climate conditions were determined based on three different

climatic models (CCSM, MIROC, and MPI) for the last glacial maximum from the Paleoclimate Modelling Intercomparison Project Phase II ($PMIP_2$; http://pmip2.lsce.ipsl.fr/).

Selected contemporary factors describe water–energy dynamics[31,32] and the importance of spatial heterogeneity[33]. These include (**Table S1**) mean annual temperature, total annual precipitation (mm×year^{-1}), Normalized Difference Vegetation Index (NDVI, unitless), and habitat heterogeneity (variability in elevation measured in m, estimated using ref.[39] elevation model).

Table S1. Historical and contemporary predictor variables used to explain spatial variation in European functional richness and dispersion. All variables were summarized for AFE grid cell (~50x50km).

Predictor variables (Units)	Estimation - Data source
Mean annual temperature velocity – [LGM to present] (km*decade^{-1})	Calculated using ref.[36] method for three models in the Paleoclimatic Modelling Intercomparison Project Phase III ($PMIP_3$; http://pmip3.lsce.ipsl.fr/) with data for the LGM. Velocity values were averaged across all models.
Annual precipitation velocity– [LGM to present] (km*decade^{-1})	Calculated using ref.[36] method for three models in the Paleoclimatic Modelling Intercomparison Project Phase III ($PMIP_3$; http://pmip3.lsce.ipsl.fr/) with data for the LGM. Velocity values were averaged across all models.
Accessibility to postglacial re-colonization from LGM refugia (km^{-1})	Calculated for a location as the summed inverse distance of each focal to all cells considered as refugia during the LGM. Refugia regions established for the LGM predictions of three models in the Paleoclimatic Modelling Intercomparison Project Phase III ($PMIP_3$; http://pmip3.lsce.ipsl.fr/) based on ref.[38] model of minimum climatic requirements for cool-temperate trees. Accessibility values were averaged across all models.
Mean annual temperature (°C)	Estimated from monthly values using the Worldclim dataset[39]
Total annual precipitation	Summed total monthly precipitation, estimated based on monthly values in the Worldclim dataset[39]
Normalized Difference Vegetation Index [NDVI] (Unitless)	Mean yearly NDVI over the 1981 to 2003 period based on the FAO Annual Sum NDVI[40].
Elevation variability (m)	Standard deviation of elevations of all cells contained within a per AFE-cell. Values extracted from the Worldclim dataset[39]

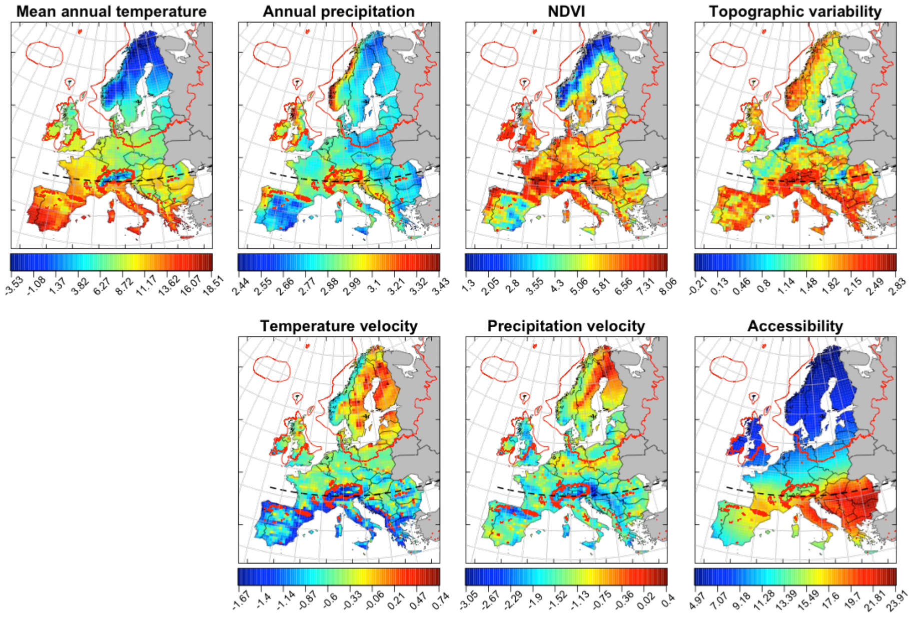

Figure S3. Contemporary and historical environmental predictors used as explanatory variables of functional richness and dispersion patterns of six orders of European angiosperms. From top to bottom and left to right: mean annual temperature, annual precipitation, NDVI, topographic heterogeneity, temperature and precipitation velocity, and accessibility to glacial refugia. Red polygon shows the maximum extent of the ice sheet 21,000 years BP. Dashed line indicated the northern limit of post-glacial refugia for cold tolerance trees as summarized in ref.[2]. Figure generated using R[3] version 3.0.2 <https://www.r-project.org> using information in the sources described in **Table S1**.

Spatially corrected pairwise correlations between variables indicated no clear pattern of the correlations between variables (**Fig. S4**). However, there was a significant negative correlation between historical and contemporary factors.

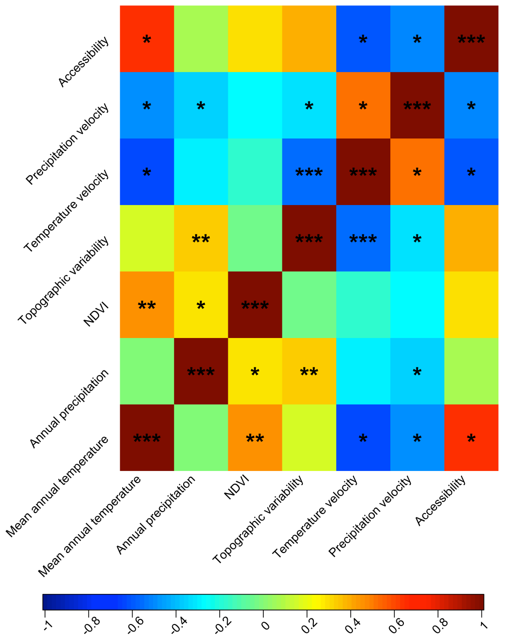

Figure S4. Pearson correlated coefficients between environmental variables used in this study. Significance of the correlation after accounting for spatial autocorrelation determined using Dutilleu-correction[41]. Significance levels: ***=P < 0.001; **=P < 0.01; *=P < 0.05; blank =not significant.

SI Text-2. **Extended results**–Association between multivariate metrics of functional diversity with both species richness and single trait range and variance.

Across all evaluated orders, as the number of species in grid accumulates the functional richness (F_{Rich}) increases **Fig. S5**. This continuous increase is contrasting to similar analyses based on all tree species in Europe[42], where F_{Rich} decelerated at richness levels below 30 species. By comparison, F_{Disp} association with species richness show multiple shapes across evaluated orders, but the most common shape of these relations is a unimodal trend (**Fig. S5**). For most of orders, F_{Disp} of those species-rich sites converges to the regional dispersion.

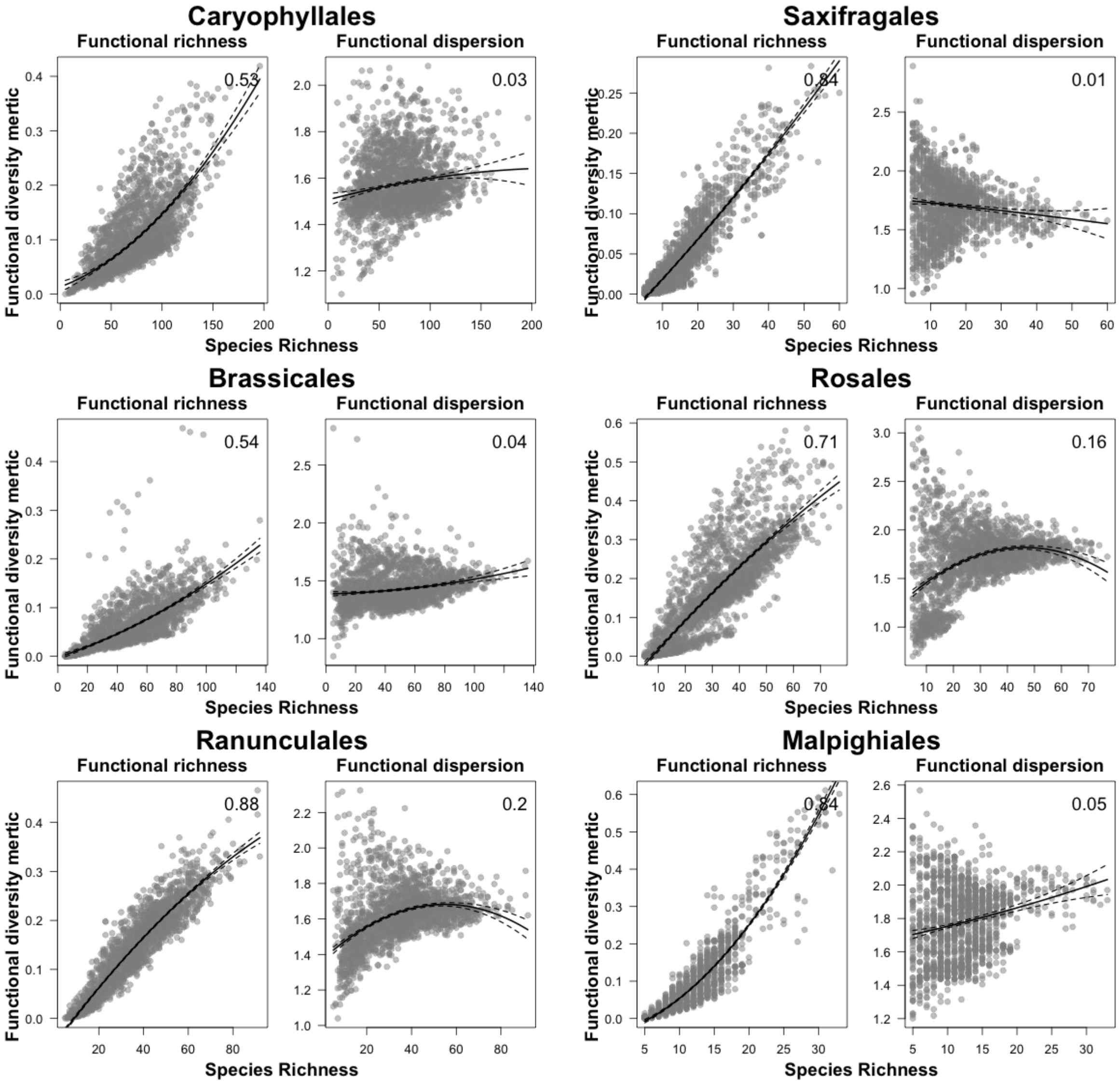

Figure S5. Scatter plots of the relation between species richness and functional richness or functional dispersion. Plotted values show the species richness and functional diversity for in each AFE grid cell, for all six evaluated orders of European angiosperms. Curves are unimodal OLS regressions between species richness and functional diversity. Explained variance (R^2) of each one of the evaluated models presented in the upper right corner of each panel.

Residuals of functional richness/dispersion vs. species richness loess regressions exhibited distinct fine scale variability (**Fig. S6**). However, regression residuals for both F_{Rich} and F_{Disp} across all orders showed a consistent south to north gradient, and a predominance of negative values (blue to light-green). This indicates a predominance of lower than expected F_{Rich} and F_{Disp} based on the number of species in a location.

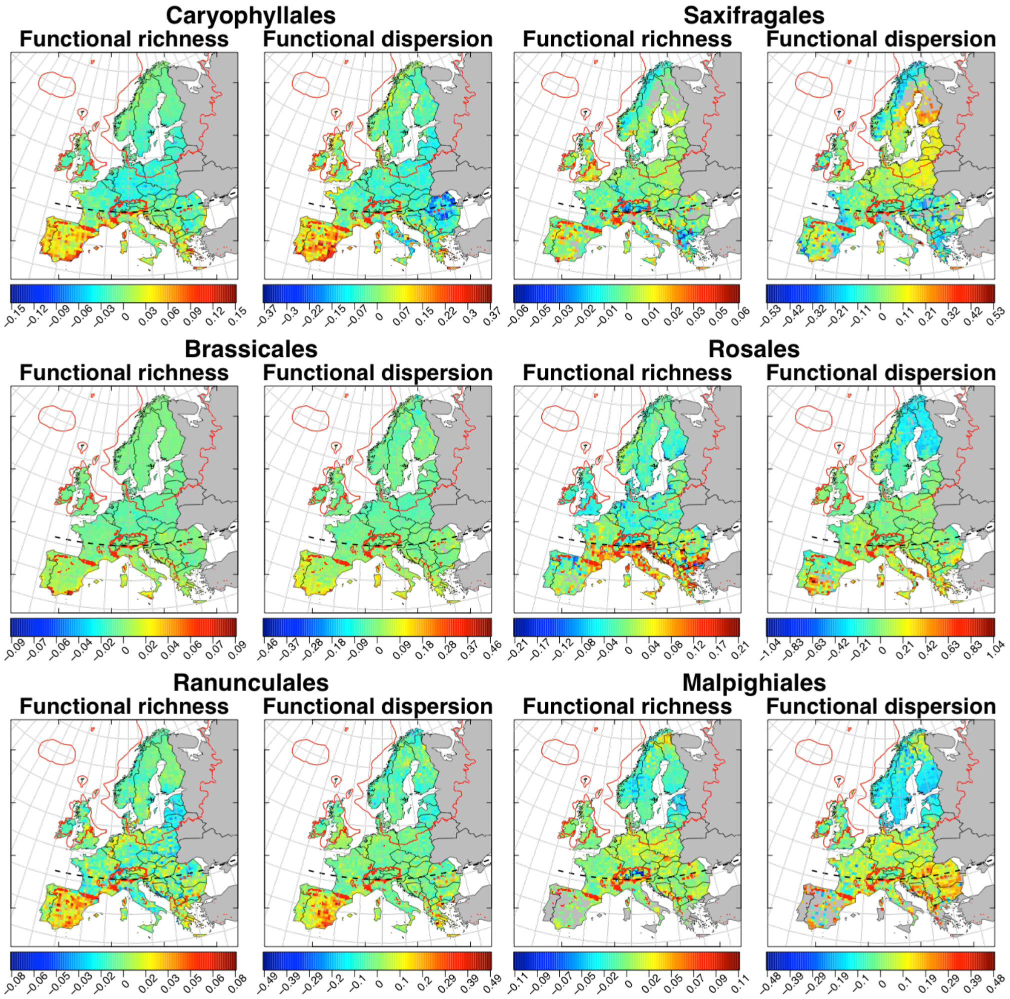

Figure S6. Residual variation of a loess-model predicting functional richness and dispersion as a function of species richness per Atlas Florae Europaeae grid cells (~50×50 km). Values show the residuals of loess-regressions per each of the Atlas Florae Europaeae grid cell (~50×50 km). Red polygon shows the maximum extent of the ice sheet during the Last Glaciation (21,000 years BP). Dashed line indicated the northern limit of post-glacial refugia for cold tolerance trees as summarized in ref.[2]. Negative residuals indicated that observed functional diversity is lower than richness-expected values, while positive residuals indicate the opposite. Figure generated using R[3] version 3.0.2. <https://www.r-project.org> based on species richness standardized estimates of functional richness and dispersion for each AFE grid cells.

The strength of the relation of F_{Rich} with trait range and of F_{Disp} with trait variability was similar across evaluated traits. This pattern is consistent across most of the evaluated orders (**Fig. S7**). Variation in F_{Rich} was strongly and positively associated with the local trait range (richness) of canopy height and seed weight for almost all of the evaluated orders (**Fig. S7**). Similarly, the variance in wood density, WD and SWT had the strongest effects on F_{Disp} for almost all of the evaluated orders (**Fig. S7**). The association of F_{Rich} with H_{max} and SWT, and of F_{Disp} with WD and SWT indicate that the trait space and its packing is maximized in areas with a wide array of dispersal strategies and growth forms.

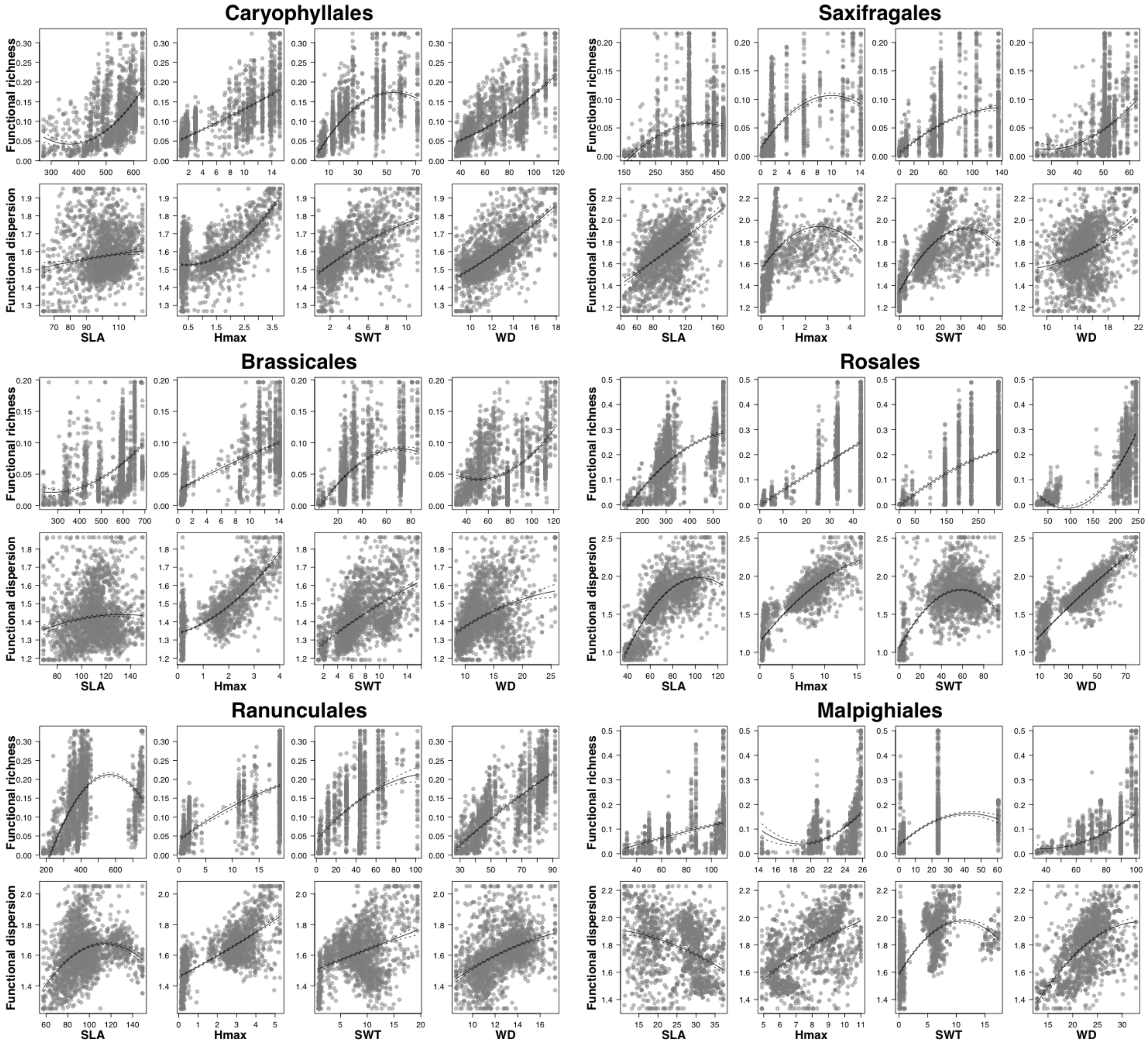

Figure S7. Scatter plots of the relation between functional richness and the trait range, and functional dispersion and trait variance for six orders of European angiosperms included in the Atlas Florae Europaeae. Curves are unimodal OLS regressions between species trait range and dispersion and functional diversity.

References

1 Jalas, J. & Suominen, J. *Atlas Florae Europaeae. Distribution of vascular plants in Europe, Vol. 1-12*. (Helsinki: Societas Biologica Fennica Vanamo, 1964-1999, 1994-1999).

2 Svenning, J.-C. & Skov, F. Could the tree diversity pattern in Europe be generated by postglacial dispersal limitation? *Ecology Letters* **10**, 453-460 (2007).
3 R: A language and environment for statistical computing. (R Foundation for Statistical Computing, Vienna, Austria, 2013).
4 Westoby, M., Falster, D. S., Moles, A. T., Vesk, P. A. & Wright, I. J. Plant ecological strategies: Some leading dimensions of variation between species. *Annual Review of Ecology and Systematics* **33**, 125-159 (2002).
5 Diaz, S. *et al.* The global spectrum of plant form and function. *Nature* **529**, 167-U173 (2016).
6 Díaz, S. *et al.* The plant traits that drive ecosystems: Evidence from three continents. *Journal of Vegetation Science* **15**, 295-304 (2004).
7 Wright, I. J. *et al.* The worldwide leaf economics spectrum. *Nature* **428**, 821-827 (2004).
8 Moles, A. T. *et al.* A brief history of seed size. *Science* **307**, 576–580 (2005).
9 Swenson, N. & Enquist, B. in *American Journal of Botany* Vol. 94 451-459 (2007).
10 Ordonez, A. & Olff, H. Do alien plant species profit more from high resource supply than natives? A trait-based analysis. *Global Ecology and Biogeography* (2013).
11 Ordonez, J. C. *et al.* A global study of relationships between leaf traits, climate and soil measures of nutrient fertility. *Global Ecology and Biogeography* **18**, 137-149 (2009).
12 Reich, P. B., Walters, M. B. & Ellsworth, D. S. From tropics to tundra: Global convergence in plant functioning. *Proceedings of the National Academy of Sciences of the United States of America* **94**, 13730-13734 (1997).
13 Falster, D. S. & Westoby, M. Plant height and evolutionary games. *Trends in Ecology & Evolution* **18**, 337-343 (2003).
14 Moles, A. T. & Westoby, M. Seed size and plant strategy across the whole life cycle. *Oikos* **113**, 91-105 (2006).
15 Leishman, M. R., Wright, I., Moles, A. T. & Westoby, M. in *Seeds: The Ecology of Regeneration in Plant Communities* (ed M. Fenner) 31–57 (CABI Publishing, 2000).
16 Chave, J. *et al.* Towards a worldwide wood economics spectrum. *Ecology Letters* **12**, 351-366 (2009).
17 Kleyer, M. *et al.* The LEDA Traitbase: a database of life-history traits of the Northwest European flora. *Journal of Ecology* **96**, 1266-1274 (2008).
18 Ordonez, A., Wright, I. J. & Olff, H. Functional differences between native and alien species: a global-scale comparison. *Functional Ecology* **24**, 1353-1361 (2010).
19 Kattge, J. *et al.* TRY – a global database of plant traits. *Global Change Biology* **17**, 2905-2935 (2011).
20 Liu, K., Eastwood, R. J., Flynn, S., Turner, R. M. & Stuppy, W. H. (2008).
21 USDA-NRCS. (ed National Plant Data Team) (Greensboro, NC 27401-4901 USA, 2013).
22 Rubin, D. B. *Multiple imputation for nonresponse in surveys*. Vol. 307 (Wiley. com, 2009).
23 Schafer, J. L. Multiple imputation: a primer. *Statistical methods in medical research* **8**, 3-15 (1999).
24 Buuren, S. & Groothuis-Oudshoorn, K. MICE: Multivariate imputation by chained equations in R. *J Stat Softw* **45**, 1-67 (2011).
25 Heitjan, D. F. & Little, R. J. Multiple imputation for the fatal accident reporting system. *Applied Statistics*, 13-29 (1991).
26 Schenker, N. & Taylor, J. M. Partially parametric techniques for multiple imputation. *Computational Statistics & Data Analysis* **22**, 425-446 (1996).

27 Horton, N. J. & Lipsitz, S. R. Multiple imputation in practice: comparison of software packages for regression models with missing variables. *The American Statistician* **55**, 244-254 (2001).
28 Cornwell, W. K., Schwilk, D. W. & Ackerly, D. D. A trait-based test for habitat filtering: Convex hull volume. *Ecology* **87**, 1465-1471 (2006).
29 Laliberté, E. & Legendre, P. A distance-based framework for measuring functional diversity from multiple traits. *Ecology* **91**, 299-305 (2010).
30 Villéger, S., Mason, N. W. H. & Mouillot, D. New multidimensional functional diversity indices for a multifaceted framework in functional ecology. *Ecology* **89**, 2290-2301 (2008).
31 Hawkins, B. A. *et al.* Energy, water, and broad-scale geographic patterns of species richness. *Ecology* **84**, 3105-3117 (2003).
32 Kreft, H. & Jetz, W. Global patterns and determinants of vascular plant diversity. *Proceedings of the National Academy of Sciences of the United States of America* **104**, 5925-5930 (2007).
33 Ricklefs, R. E. Community diversity: relative roles of local and regional processes. *Science* **235**, 167-171 (1987).
34 Svenning, J.-C., Eiserhardt, W. L., Normand, S., Ordonez, A. & Sandel, B. The Influence of Paleoclimate on Present-Day Patterns in Biodiversity and Ecosystems. *Annual Review of Ecology, Evolution, and Systematics* **46** (2015).
35 Normand, S. *et al.* Postglacial migration supplements climate in determining plant species ranges in Europe. *Proc Biol Sci* **278**, 3644-3653 (2011).
36 Sandel, B. *et al.* The influence of late Quaternary climate-change velocity on species endemism. *Science* **334**, 660-664 (2011).
37 Svenning, J.-C. *et al.* Geography, topography, and history affect realized-to-potential tree species richness patterns in Europe. *Ecography* **33**, 1070-1080 (2010).
38 Leroy, S. A. G. & Arpe, K. Glacial refugia for summer-green trees in Europe and south-west Asia as proposed by ECHAM3 time-slice atmospheric model simulations. *Journal of Biogeography* **34**, 2115-2128 (2007).
39 Hijmans, R., Cameron, S., Parra, J., Jones, P. & Jarvis, A. Very high resolution interpolated climate surfaces for global land areas. *International Journal of Climatology* **25**, 1965-1978 (2005).
40 FAO. (FAO, Rome, Italy, 2014).
41 Dutilleul, P., Clifford, P., Richardson, S. & Hemon, D. Modifying the t test for assessing the correlation between two spatial processes. *Biometrics* **49**, 305-314 (1993).
42 Swenson, N. G. *et al.* Constancy in Functional Space across a Species Richness Anomaly. *American Naturalist* **187**, E83-E92 (2016).
